# Ultra-wideband filtering of spoof surface plasmon polaritons using deep subwavelength planar structures

**DOI:** 10.1038/srep37605

**Published:** 2016-11-24

**Authors:** Ming Zhe Hu, Hao Chi Zhang, Jia Yuan Yin, Zhao Ding, Jun Feng Liu, Wen Xuan Tang, Tie Jun Cui

**Affiliations:** 1 Department of Physics and Electronics, Liupanshui Normal University, Liupanshui, Guizhou 553004, China; 2 State Key Laboratory of Millimeter Waves, Southeast University, Nanjing 210096, China; 3 College of Big Data and Information Engineering, Guizhou University, Guiyang, Guizhou 550025, China

## Abstract

Novel ultra-wideband filtering of spoof surface plasmon polaritons (SPPs) is proposed in the microwave frequency using deep subwavelength planar structures printed on thin and flexible dielectric substrate. The proposed planar SPPs waveguide is composed of two mirror-oriented metallic corrugated strips, which are further decorated with parallel-arranged slots in the main corrugated strips. This compound structure provides deep subwavelength field confinement as well as flexible parameters when employed as a plasmonic waveguide, which is potential to construct miniaturization. Using momentum and impedance matching technology, we achieve a smooth conversion between the proposed SPPs waveguide and the conventional transmission line. To verify the validity of the design, we fabricate a spoof SPPs filter, and the measured results illustrate excellent performance, in which the reflection coefficient is less than −10 dB within the −3 dB passband from 1.21 GHz to 7.21 GHz with the smallest insertion loss of 1.23 dB at 2.21 GHz, having very good agreements with numerical simulations. The ultra-wideband filter with low insertion loss and high transmission efficiency possesses great potential in modern communication systems.

Surface plasmon polaritons (SPPs) are a kind of surface electromagnetic (EM) waves inspired by the coupling between free electrons in a metal and incident EM waves. The incoming EM fields will be confined tightly on the metal/dielectric interface and the electric energy will be greatly enhanced in a subwavelength scale with good modal shape and low propagation loss^1^,^2^,^3^. Due to this characteristic, SPPs have propelled great research interests for potential applications in high sensitive biochemical sensors^4^, super-resolution imaging^5^,^6^, and miniaturized photonic circuits^7^. However, natural SPPs effect can only be considered for applications at optical frequency since the intrinsic electron oscillation in a metal is usually located beyond the infrared band^8^. While at much lower frequency band, such as microwave and terahertz frequencies, deep subwavelength effect of SPPs cannot be realized due to the disability of internal plasmonic oscillations in metal.

This problem has not been solved until 2004, when Pendry *et al*. put forward a concept of spoof SPPs to produce deep subwavelength effect at microwave and terahertz frequencies in metallic structures^9^. After that, various kinds of artificial periodical patterns, such as one- dimensional (1D) chain of slots^10^,^11^,^12^,^13^,^14^, two-dimensional (2D) mushroom-like metallic surfaces^15^, and even bending artificial plasmonic structures^16^,^17^ have been proposed and fabricated for controlling and steering EM signals in microwave and terahertz regimes in recent years. These spoof plasmonic structures are usually composed of periodically patterned metallic structure in millimeter scale size on ultralow loss dielectrics. They possess not only similar capacity of field confinement and non-diffraction limit as that of optical SPPs, but also the dispersion property with cutoff frequency of nature SPPs. Additionally, these spoof SPPs can be conveniently and flexibly tailored by optimizing the geometrical parameters on patterns due to their millimeter scale size, much larger than the nanoscale size applied in optical SPPs. With these advantages, it is promising that spoof SPPs have profound significance for continuously spurring the development of compact, ultrafast and low-power digital circuitries at microwave and terahertz frequencies.

In this paper, we firstly propose a novel structure for spoof SPPs at microwave frequencies. Based on the structure, we design and fabricate an ultra-wideband filter with low reflection and high transmission coefficient for SPPs waves. The spoof SPPs waveguide is composed of mirror-oriented corrugated metallic strips, in which compound slot geometry is further designed. The compound slot structure is composed of mother slots and son slots, where the son slots are parallel and symmetrically arranged on the two sides of the mother slots, as can be referred to the inset of Fig. 1. Using the presented artificial plasmonic waveguide, we can confine the microwave energy tightly with little propagation loss. Also, in order to reach a perfect momentum matching between the spoof SPPs waveguide and the signal input port, where a traditional co-planar waveguide (CPW) working in quasi TEM mode is employed, a transition section with gradient slots and flaring ground is designed for high-efficiency conversion. Numerical simulations and experimental results show that the presented plasmonic waveguide owns high efficiency filtering of spoof SPPs in ultra-wide frequency band, which builds a solid avenue for large-scale plasmonic integrated circuits in microwave and terahertz devices.

## Results

### The designed compound slots structure for plasmonic waveguide

**Figure 1:**
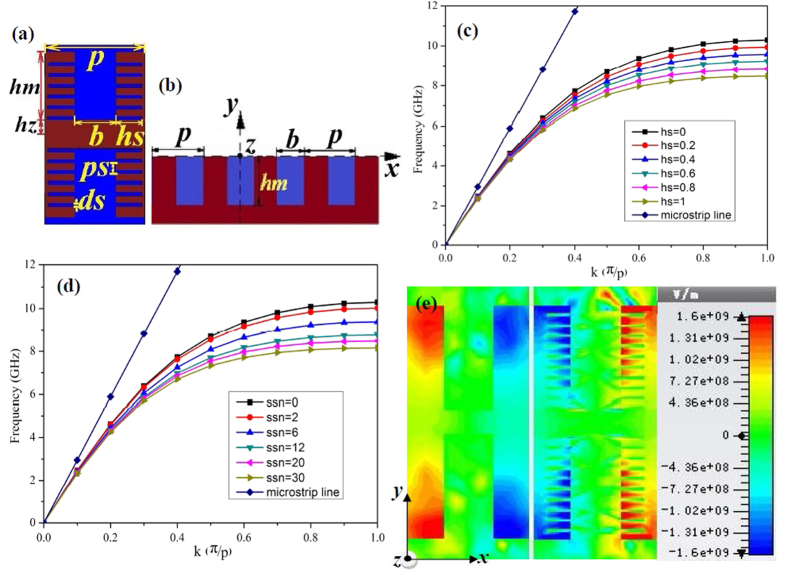
(**a**) The schematic unit cell of the spoof SPPs waveguide, which is composed of compound slot structure (mother slot and son slot) and in this sample the son slot number is 12. (**b**) The schematic of one dimensional groove array with the depth of *hm*, the width of *b* and the lattice constant of *p* on a perfect metal surface with an infinite thickness along the *z* direction. (**c**) The dispersion diagrams of the spoof SPPs structure with different son slot depths *hs* (with the son slot number being 20, the son slot width *ds* being 0.1 mm, the son slot period *ps* being 0.35 mm, the mother slot depth *hm* being 4.5 mm, the mother slot width *b* being 2 mm and the mother slot period *p* being 5 mm). (**d**) The dispersion diagrams of the spoof SPPs structure with different son slot numbers *ssn* (with the son slot depth *hs* being 1.0 mm, *ds* being 0.1 mm and the parameters of the mother slot being the same as that in (**c**)). (**e**) Represents the EM confinement (simulated) in the H-shaped mother slot (*hs* = 0, left) and in the compound slot with *hs* = 1.0 and *ssn* = 20 (right).

The corrugated metal with compound slots structure is printed on a 0.5 mm thick dielectric substrate F4B with dielectric constant ε_r_ = 2.65 and loss tangent tanδ = 0.003 at microwave frequencies. The metal is chosen as annealed copper with film thickness of 0.018 mm. The thickness of dielectric substrate and copper film correspond to 167/10000 and 6/10000 of the EM wavelength in free space at 10 GHz respectively. Figure 1 depicts the dispersion and E-filed distribution characteristics of the proposed artificial plasmonic structure. Figure 1(a) shows the corrugated metallic unit cell is composed of two sets of periodic slots, where the depth, width and period of the main slot (here we call it mother slot) are denoted as *hm, b*, and *p*, respectively. On the two sides of the mother slots, there distributes parallel, periodical and mirror symmetric son slots. The son slots are designed to enhance the equivalent capacitance and inductance of the mother slot. The geometrical parameters of son slots are denoted as periodic *ps*, depth *hs* and width *ds*, respectively, in which, the periodic *ps* varies with the son slot number (denoted as *ssn*) so as to guarantee the even distribution of son slots in the mother slot. According to R. F. Harrington^18^, for one dimensional groove array with the geometry parameters of the above mother slot, but being infinitely thick in *z* direction, as illustrated in Fig. 1(b), when the incident EM wave is p-polarized, the wave impedance looking into the corrugated surface in the negative *y* direction is:


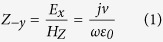


where


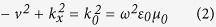


in which, *k*_*x*_ is the wave number in the *x* direction of the surface of F4B substrate and *k*_*0*_ is the wave umber in free space. On the other side, the parallel-plate transmission-line mode exists in the short-circuited slots of the metal corrugation, thus, the input wave impedance from the negative *y* direction can be described as ref. 19:


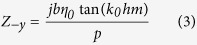


where, 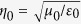 is the intrinsic impedance of free space. When EM wave resonates at the interface of metal corrugate, we can deduce Eq. (4) by equating Eqs (1)~(3) as following:


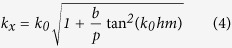


From Eq. (4), one can clearly infer that when the slot width *b* increases, the wave number *k*_*x*_ will be enhanced as well. Inspired by this, we design the corrugated son slots on the two sides of mother slots with finite film thickness to expect the analogous physical effects. Meanwhile, for symmetrical transmission, the above groove array has been designed mirror-oriented along it propagation direction. It is anticipated that the increase of the son slot depth *hs* will augment the wave number *k*_*x*_ as well as the input wave impedance *Z*_*−y*_ of the mother slots (refer to Eq. (3)) in the present model.

To verify the above designing theory, all the numerical simulations of the compound slots have been carried out by the commercial software, CST Microwave Studio. Results show that the dispersion curve deviates more quickly and the equivalent cutoff plasmonic frequency reduces with the increase of son slot depth *hs* from 0 to 1.0 mm (whilst the son slot width *ds* is fixed at 0.1 mm during the numerical simulation), as illustrated in Fig. 1(c), where *hs* = 0 corresponds to the dispersion curve of the previously proposed metallic corrugated strips by Ma *et al*.^20^. These results correspond quite well with the above designing theory and this unique feature much resemble the behavior that originally happen in optical frequencies of the natural SPPs, which will result in a smaller propagating wavelength and a tighter electric field confinement of the EM wave at gigahertz frequency, ensuring reliable applications in miniaturized plasmonic devices and low-interference SPPs circuits^21^. Also, as the son slot number increases from 0 to 30 (with the son slot depth *hs* being 1.0 mm), we find a similar dispersion curve with lower asymptotical cutoff frequencies as that in the increase of son slot depth, as depicted in Fig. 1(d). These dispersion curves with lower cutoff frequencies will lead to larger propagating vectors in the plasmonic waveguides, which indicates the proposed spoof SPPs provides more tailoring parameters than the previously reported corrugated metallic structure^20^ and allows easier tuning of the wave momentum and propagating vector in plasmonic circuits. Also, the field confinement effect is much more significant in compound slots structure than that in ref. 20 as indicated by Fig. 1(e). In this regard, the proposed structure allows more flexible and convenient application in plasmonic circuit industry without increasing the size or cost of devices.

### Plasmonic wide bandwidth filter with transition section

**Figure 2:**
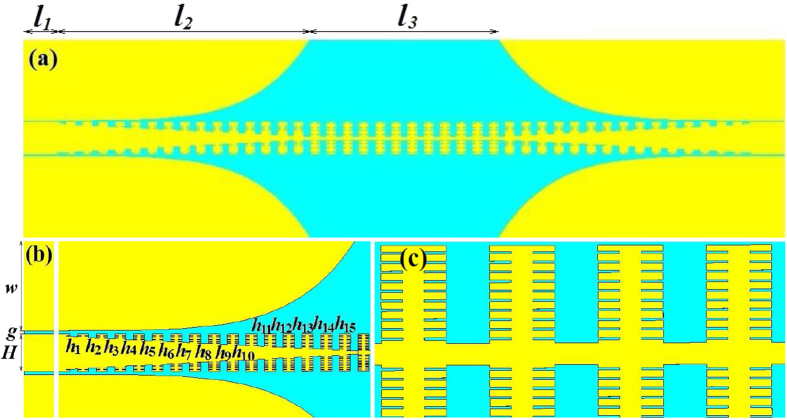
The simulated structure of the particular spoof SPPs filter. (**a**) Proposed filter composed of three sections with the length of each part being *l*_*1*_*, l*_*2*_ and *l*_*3*_ respectively. (**b**) The CPW section and the transition section with the gradually varying slots denoted as *h*_*i*_ (*i* = 1~15), and (**c**) the spoof SPPs waveguide section.

By employing the proposed spoof SPPs waveguide, we designed an ultra wideband microwave filter, whose structure is formed by three sections printed on the top surface of the substrate, whose lengths are *l*_*1*_ = 8 mm, *l*_*2*_ = 80 mm and *l*_*3*_ = 58 mm respectively, as depicted in Fig. 2. In the input and output sections, conventional CPWs are employed to feed microwave signal or to receive the transmitted signal. The middle section of the filter is composed of the proposed mirrorly oriented corrugated metallic strip with the compound slot structure. It has been analyzed above that the compound slot structure is a good plasmonic waveguide with tight EM field confinement and small transmission loss. However, this plasmonic waveguide cannot be directly integrated with the first CPW section because the quasi TEM mode in the latter is mismatched in wave momentum with the spoof SPPs mode in the former.

Previously reported methods such as dipole antenna method^21^,^22^, prism method^23^ and gratings method^24^,^25^ have been put forward to connect SPPs modes with quasi TEM modes. However, the relatively low conversion efficiency due to momentum mismatch between them have prevented these methods from industrial applications. Later, a high efficiency transition waveguide between the quasi TEM transmission line and the spoof SPPs line was proposed in ref. 26, which is composed of a linearly gradient corrugated metallic strip and an exponential flaring curve ground. The mixed modes of quasi-TEM and spoof SPPs are simultaneously supported on the transition section, where quasi-TEM mode dominates in the beginning and is gradually converted to the designed plasmonic mode as the groove depth increases step by step until it turns into the pure spoof SPPs mode at last. Although the transition efficiency is significantly increased through this design, it can still be improved through modifying the flaring ground curve, which is actually a crucial point to provide gradient momentum compensation and impedance matching between quasi-TEM modes and plasmonic TM modes. Actually, we have modified the flaring ground curve using several mathematical functions in the present work as will be discussed later in this paper, and we found a better transition effect than that given in ref. 27.

**Figure 3:**
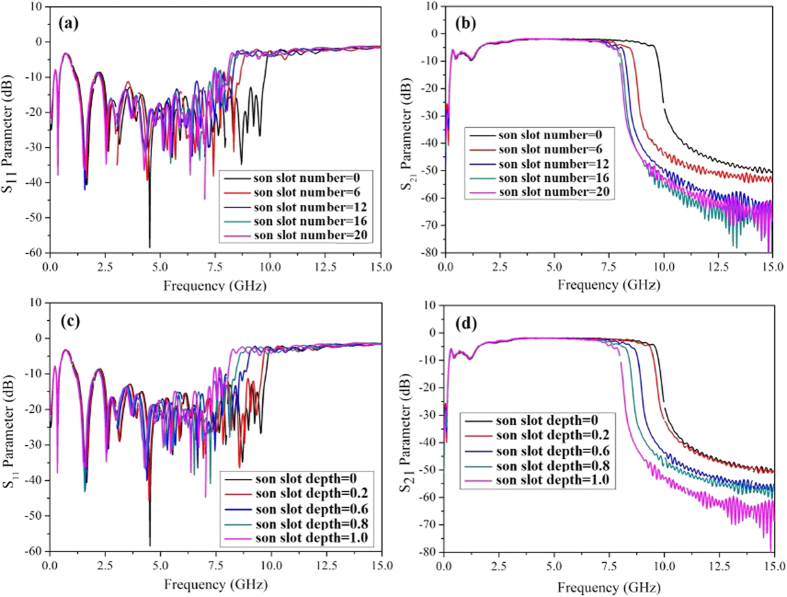
Simulated scattering parameters of the presented spoof SPPs filter at microwave frequencies. (**a**) S_11_ and (**b**) S_21_ vary with the son slot number. (**c**) S_11_ and (**d**) S_21_ vary with the son slot depth (with the son slot number being 20).

After designing the structure of the particular filter, we simulated the scattering parameters (S_11_ and S_21_) from 0 to 15 GHz and the results are illustrated in Fig. 3. The geometrical parameters for the designed filter are as follows: the width of the ground w = 25.0 mm, the gap in CPW section g = 0.4 mm and the width of the transmission line in CPW section H = 5.0 mm. The depth of the mother slot linearly increases from 0.25 mm to 4.5 mm with the period being 15 and the step width being 0.2833 mm in the transition section, while the son slot number and its depth is fixed at 20 and 1.0 mm respectively in this section.

It is observed that the spoof SPPs waveguide is capable of EM wave transmission from almost DC frequency to the cutoff gigahertz frequency with high transmission and low reflection, as illustrated in Fig. 3. The upper stop band of the spoof SPPs filter can be artificially adjusted by the geometrical dimensions of the son slot, such as the son slot number and the son slot depth. As can be seen, with the increase of son slot number or son slot depth, the upper stop band frequency decreases and the stop band becomes steeper, which means the filter possesses better out-of-band properties. Especially, the simulated upper stop band frequency decreases almost linearly from 9.03 GHz to 7.25 GHz with the increase of son slot depth from 0 to 1.0 mm, which provides a convenient and precise control of the stop frequency for the filter. Note that these adjustment is very flexible and without any increase of filter size or cost.

At lower stop band frequency, the S-parameter curves are rarely adjusted by the geometrical parameters of the son slot. In the whole 3 dB passband the reflection is less than −10 dB and the transmission curve is flat with ripples of less than 1.5 dB. However, near the upper and lower stop frequencies, the reflection ripple becomes higher, resulted from the momentum mismatch at these frequency ranges. The mismatch can actually be improved by tailoring the shape and length of the transition section. In terms of this, we deduce that the transition section is a key part to optimize the operation properties of the spoof SPPs filter. To more accurately and quantitatively evaluate the transmission properties of the presented spoof SPPs structure, we calculate the transmission loss in the spoof SPPs mode section, where the influence of the transition sections at both ends are extracted. Result shows that the transmission loss is only 0.02 dB/cm at 7 GHz and it decreases along with the frequency. For comparison, we have also calculated the transmission loss in a conventional microstrip line with the same length and strip width as the spoof SPPs waveguide in Fig. 2. It is found that although the propagation loss of the presented spoof SPPs structure is twice larger that of conventional microstrip, which is about 0.01 dB/cm at 7 GHz, the former provides an approach for the convenience of integrated circuits with its single-side conductor feature and low crosstalk property. In addition, the simulated relative bandwidth of the filter with son slot number being 20 and son slot depth being 1.0 mm can reach 117%, manifesting itself an ultra-wideband filter that can be utilized in wideband high speed data communication.

### The influence of the flaring ground curve

**Figure 4:**
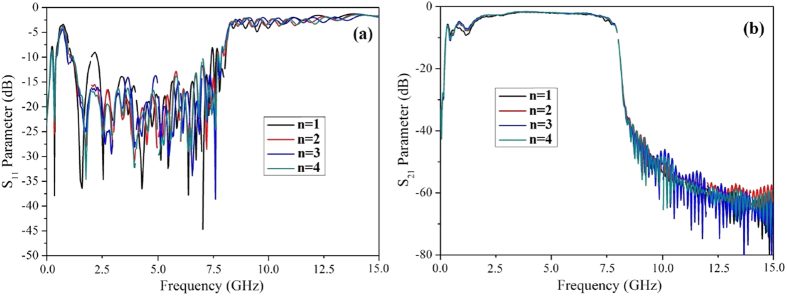
Simulated scattering parameters of the presented spoof SPPs filter varied with the ground curve shape parameter n at microwave frequencies. (**a**) S_11_ parameter and (**b**) S_21_ parameter.

As has been mentioned above, it is worthy to note that the flaring ground curve in the transition section is very important for high efficient transmission and minimum reflection loss of the spoof SPPs filter. Thus, we have designed several kinds of flaring ground using different mathematical functions, including circle function, parabola function and exponential function for comparison. Results show that the exponential one is the optimal function for highly efficient EM modes conversion. Also, the shape of the exponential curve is critical for the highly efficient signal transmission. Here, we define the exponential curve as:


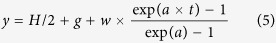


and


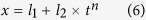


where *n* is the shape parameter that can control the flaring speed of the exponential curve. As can be seen in Fig. 4, with the variation of n (n ≥ 1), the reflection and transmission characteristics of the filter can be obviously improved, especially at the frequency close to the lower stop band of the S_11_ curve, which means that the exponential curve shape contributes greatly to the momentum conversion in the pass band of the filter. However, as the parameter *n* increases to 4, the reflection energy increases again, particularly at the frequency close to the upper stop band of the S_11_ curve. Therefore, n = 3 is the optimal shape curve parameter for the presented transition section.

### Fabrication and measurement of the spoof SPPs filter

**Figure 5:**
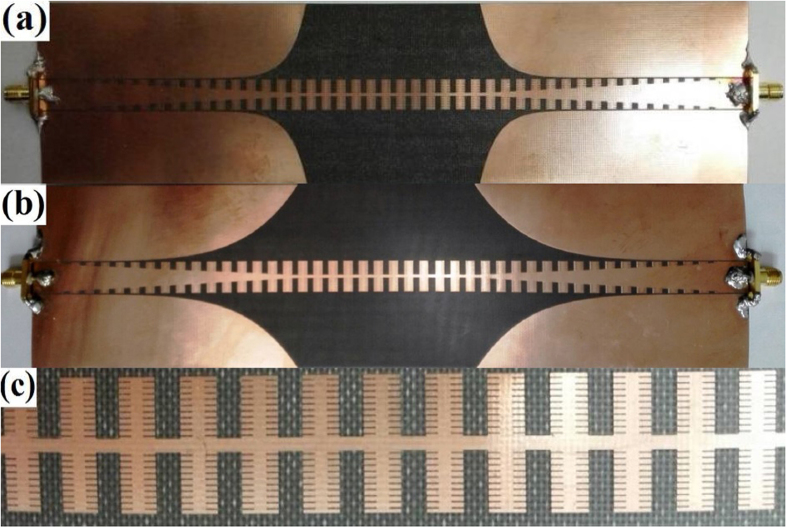
Synthesized spoof SPPs filter with son slot number being 20. (**a**) Shape parameter n = 1. (**b**) Shape parameter n = 3. (**c**) Detail of the spoof SPPs section.

**Figure 6:**
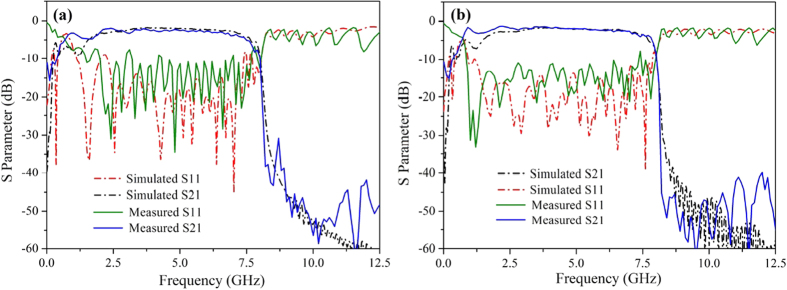
Measured and simulated S-parameters of the presented SPPs filter with the exponential curve shape parameter of (**a**) n = 1 and (**b**) n = 3.

In order to verify the design and simulation, we fabricate the above spoof SPPs filter by traditional PCB printing method, as illustrated in Fig. 5. The production geometry parameters are the same as the optimal parameters in simulation. Two samples with the exponential curve parameter n = 1 and n = 3 were synthesized for comparison. The S-parameter of the synthesized filter was measured by Agilent vector network analyzer (VNA, N5230C). The measured and simulated results of the S-parameters, including the reflection coefficients S_11_ and transmission coefficients S_21_, are illustrated in Fig. 6. It is obvious that there is a good agreement between the measured and simulated S-parameters, especially for the two S_21_ curves. Moreover, the measured S_21_ parameter is even better than the simulated one at lower frequencies. The measured S_11_ parameter, however, is worse than the simulated one, which can be ascribed to the impedance mismatch at two welding end points. The transmission coefficient S_21_ indicates good frequency-selective property of the proposed structure with the transmission loss being low and companied by a transmission zero at 0.21 GHz, which helps to suppress the low-frequency interference. The reflection coefficient S_11_ of the n = 3 sample is much better than that of the n = 1 sample, which is in good agreement with the simulated result in Fig. 4. In the whole pass band from 1.21 GHz to 7.21 GHz, the S_11_ parameter of the n = 3 sample is less than −11.2 dB, which manifests the good impedance and momentum matching behavior from the CPW waveguide to the SPPs waveguide through the presented exponential curve ground.

### Field confinement effects

**Figure 7:**
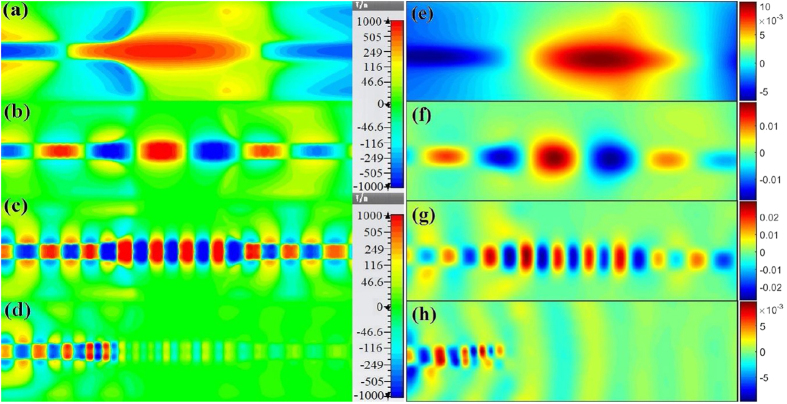
The simulated and measured near electric field (E_z_ component) of the presented filter at different frequencies. (**a**–**d**) The simulated E_z_ distributions at 1 GHz, 3 GHz, 7 GHz and 10 GHz. (**e**–**h**) The measured E_z_ distributions at 1 GHz, 3 GHz, 7 GHz and 10 GHz.

To get a direct physical insight into the mode matching transition, as well as the properties of field propagation and confinement on the spoof SPPs waveguide with compound slot structure, we performed full-wave simulations using commercial CST Microwave Studio. Figure 7 demonstrates the energy flows (on a dB scale) toward x direction on the xoy plane that is 1.5 mm above the plasmonic surface of the waveguide. We monitored and measured four different frequencies at 1 GHz, 3 GHz, 7 GHz and 10 GHz for the sample of the son slot number being 20 and the exponential shape coefficient n being 1. It is also verified that the measured and simulated EM fields correspond quite well with each other, both in magnitude and distribution size. It is obviously evidenced the quasi TEM mode in CPW waveguide is smoothly converted to the SSPPs mode with low reflection. The EM energy is tightly confined in deep subwavelength scale around the plasmonic waveguide and it propagates with little reflection and low absorption and radiation loss in the whole pass band from 1.21 GHz to 7.21 GHz. Moreover, the significant Ex component is detected on the plasmonic waveguide due to the transverse magnetic behavior of its eigenmodes.

## Discussion

In the present paper, we have proposed a compact spoof SPPs structure with compound corrugated metallic slots to produce frequency selective microwave filter. The compound slots structure is composed of the so called mother slot and son slot, orthogonally arranged to each other, to obtain better spoof SPPs properties. Besides the spoof SPPs waveguide using the compound slot structure, we also employ a transition waveguide to smoothly covert the quasi TEM mode to spoof SPPs mode, so as to feed and receive electromagnetic signal with low energy reflection. Plentiful numerical simulations and experiments have been employed to validate the designing theory and the transmission properties of the proposed filter. And both the simulated and measured S-parameters demonstrate that the proposed spoof SPPs structure has high efficiency transmissions (with transmission loss being only 0.02 dB/cm at 7 GHz) and low reflections loss (measured below −11.2 dB) in the designed frequency band from 1.21 GHz to 7.21 GHz. The simulated and measured near field distributions indicate that the EM energy is tightly confined in deep subwavelength scale around the spoof SPPs waveguide. Such unique performance endows the proposed spoof SPPs band-pass filter a very encouraging future in high compact microwave or even terahertz wave integrated circuits and plasmonic functional devices.

## Methods

All numerical simulations including SPPs filters and E-field distributions are conducted by the commercial software, CST Microwave Studio. The experimental structure is fabricated using a 0.5 mm thickness dielectric film, F4B, which is a kind of Teflon woven, composed of polytetra-fluoroethylene and glass fiber with its relative permittivity being 2.65 and tangent loss being 0.003 at microwave frequency. The patterned copper conductor film is printed onto the F4B substrate for the SPPs filtering with its thickness of 0.018 mm. We employ Agilent vector network analyzer (VNA, N5230C) to measure the S parameters of the SPPs filters, including the reflection coefficients S_11_ and transmission coefficients S_21_. The near E-field distributions along the z-direction of the SPPs filter are examined by a home-made near-field scanning system, where the testing antenna probe linearly scans in the xoy plane, 1.5 mm above the surface of the fabricated SPPs filter.

## Additional Information

**How to cite this article**: Hu, M. Z. *et al*. Ultra-wideband filtering of spoof surface plasmon polaritons using deep subwavelength planar structures. *Sci. Rep.* **6**, 37605; doi: 10.1038/srep37605 (2016).

**Publisher’s note:** Springer Nature remains neutral with regard to jurisdictional claims in published maps and institutional affiliations.
